# Iron(III)-Salophene: An Organometallic Compound with Selective Cytotoxic and Anti-Proliferative Properties in Platinum-Resistant Ovarian Cancer Cells

**DOI:** 10.1371/journal.pone.0002303

**Published:** 2008-05-28

**Authors:** Thilo S. Lange, Kyu Kwang Kim, Rakesh K. Singh, Robert M. Strongin, Carolyn K. McCourt, Laurent Brard

**Affiliations:** 1 Molecular Therapeutics Laboratory, Program in Women's Oncology, Department of Obstetrics and Gynecology, Women and Infants' Hospital of RI, Warren Alpert Medical School of Brown University, Providence, Rhode Island, United States of America; 2 Department of Biology and Medicine, Brown University, Providence, Rhode Island, United States of America; 3 Department of Chemistry, Portland State University, Portland, Oregon, United States of America; University of Minnesota, United States of America

## Abstract

**Background:**

In this pioneer study to the biological activity of organometallic compound Iron(III)-salophene (Fe-SP) the specific effects of Fe-SP on viability, morphology, proliferation, and cell-cycle progression on platinum-resistant ovarian cancer cell lines were investigated.

**Methodology/Principal Findings:**

Fe-SP displayed selective cytotoxicity against SKOV-3 and OVCAR-3 (ovarian epithelial adenocarcinoma) cell lines at concentrations between 100 nM and 1 µM, while the viability of HeLa cells (epithelial cervix adenocarcinoma) or primary lung or skin fibroblasts was not affected. SKOV-3 cells in contrast to fibroblasts after treatment with Fe-SP revealed apparent hallmarks of apoptosis including densely stained nuclear granular bodies within fragmented nuclei, highly condensed chromatin and chromatin fragmentation. Fe-SP treatment led to the activation of markers of the *extrinsic* (Caspase-8) and *intrinsic* (Caspase-9) pathway of apoptosis as well as of executioner Caspase-3 while PARP-1 was deactivated. Fe-SP exerted effects as an anti-proliferative agent with an IC_50_ value of 300 nM and caused delayed progression of cells through S-phase phase of the cell cycle resulting in a complete S-phase arrest. When intra-peritoneally applied to rats Fe-SP did not show any systemic toxicity at concentrations that in preliminary trials were determined to be chemotherapeutic relevant doses in a rat ovarian cancer cell model.

**Conclusion/Significance:**

The present report suggests that Fe-SP is a potent growth-suppressing agent *in vitro* for cell lines derived from ovarian cancer and a potential therapeutic drug to treat such tumors *in vivo*.

## Introduction

Minerals and metals have been employed in various forms of medical treatment for several thousand years. In ancient Egypt and Greece, in Ayurvedic medicine, asian medicine, or by the Aztecs metals in elementary form, as salts or as pharmaceutically active compounds from plants were used mainly due to anti-inflammatory effects associated with the application [1], [2]. Metals found their revival in pharmacological efforts during the renaissance [2], however, often with toxic side-effects due to heavy metal use. The earliest report on the therapeutic use of metals or metal containing compounds not only as before in ulcerous conditions but in cancer and leukemia date back to the sixteenth century [3], [4]. Several recent reviews have described the characteristics of diverse metal compounds and their use and/or putative mode of action in modern cancer treatment in pre-clinical and clinical studies [4], [5], [6].

The current treatment of a variety of tumors, including ovarian cancer, relies on organometallic platinum compounds. In the United States, epithelial ovarian cancer (EOC) is the leading cause of death from gynecologic malignancies and the fourth most common cause of death due to cancer among women [7]. An estimated twenty-two thousand new cases and an estimated fifteen-thousand deaths secondary to ovarian cancer occurred in the year 2007 [8]. Although most patients initially respond to cytoreductive surgery and adjuvant paclitaxel and platinum-based chemotherapy, the majority will experience disease recurrence. While re-treatment with a platinum-based drug is possible for some women the response rate to current second line chemotherapy is 15–30% due to the rise of resistance to such drugs requiring the development of new drugs to treat such tumors [6], [9].

**Figure 1:**
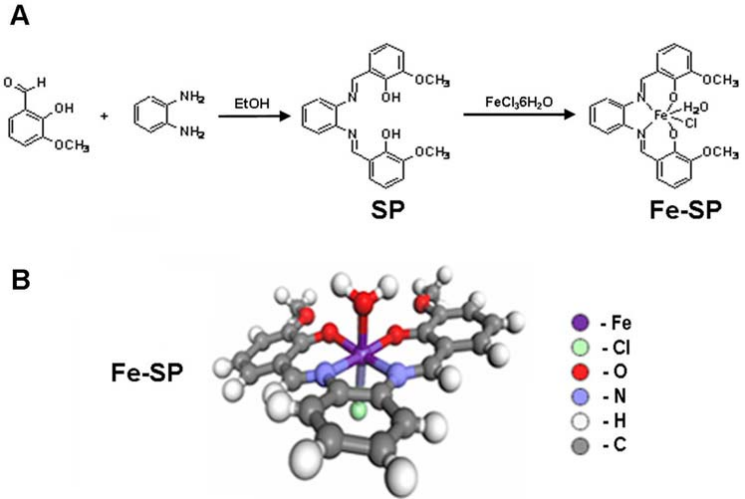
Synthesis and structure of Iron(III)-salophene. (A) Synthesis of the salophene ligand (SP; from 1,2-phenylenediamine and o-vanillin) and of the Iron(III)-salophene complex (Fe-SP) (see Materials and Methods). (B) Structural characterization of Fe-SP by X-ray crystallography (see Table 1.)

The design of new metal-based drugs is often challenged by physio-chemical properties such as insufficient solubility or hydrolytic instability making it problematic to control their delivery, stability and ultimately their specific effects *in vivo*. One approach to control the cytotoxic responses of new metal-based compounds is to engage biologically essential transition metals, such as iron (Fe), manganese (Mn), zinc (Zn), copper (Cu), or cobalt (Co) of different oxidative states and their reactive intermediates [4], [5]. The current report describes the selective cytotoxic effects of a novel organometallic complex (Iron(III)-salophene; Fig. 1A) on platinum-resistant ovarian cancer cells.

Salophenes represent a class of organic compounds defined by two Schiff's bases connecting three aromatic moieties. The two outer aromatic moieties typically feature salisaldehydes and the central aromatic moiety an *o*-phenylene-diamine or analog. Salophenes display potent binding with transition metal elements and are closely related to salens containing Schiff's bases that are constituted of aliphatic diamines. Metallosalens fall into a number of non-platinum metal compounds such as thiosemicarbazone or hydrazone pharmacophores with putative anticancer activities [4], [10]. Metallosalophenes have not been studied extensively with respect to applications in biological systems: previously salophene-lanthanide complexes were described to selectively bind to neutral sugars and lipids including lysophosphatidic acid, which serves as a marker for several pathological conditions including ovarian cancer [11] and Mn-salophene (EUK178) as well as various Mn-salens display cyto-protective features in fibroblast cultures via hydrogen peroxide scavenging [12]. A related compound (cobalt-3,4-diarylsalen) has been shown to reduce the viability of various cancer cell lines at rather high concentrations (≥20 µM) and it was suggested that the mode of activity was not linked to oxidative DNA damage [13]. Based on the present report and further studies on the selective cytotoxic effects of salophenes when complexed with transition metals, a patent was filed. This invention comprises of the synthesis, biological evaluations, applications, and pharmaceutical compositions of metal-salophenes (MSPs). Furthermore, the invention comprises of use of MSPs as drugs with therapeutic anti-neoplastic, anti-angiogenic and anti-cancer activities, and with other properties such as free radical scavenging. In addition, this invention provides methods to be applied in chemoprevention of chemical carcinogenesis and alterations of drug metabolism involving the epoxides or free oxygen radicals or intermediates.

The objective of the present study was to investigate the potential of Fe-SP as a biologically active drug in an ovarian cancer cell model with respect to dose-dependent and specific effects on viability, morphology, proliferation, and cell-cycle progression. In addition to describing the selective cytotoxicity of Fe-SP on platinum-resistant ovarian cancer cells we conducted a study on the systemic toxicity of Fe-SP when applied in rats as a model system. The present report suggests that this novel organometallic compound displays properties as a potential therapeutic drug and an alternative to platinum reagents in the treatment of ovarian cancer.

## Results

### Specific cytotoxic effect of Fe-SP on human platinum-resistant ovarian cancer cell lines

**Figure 2:**
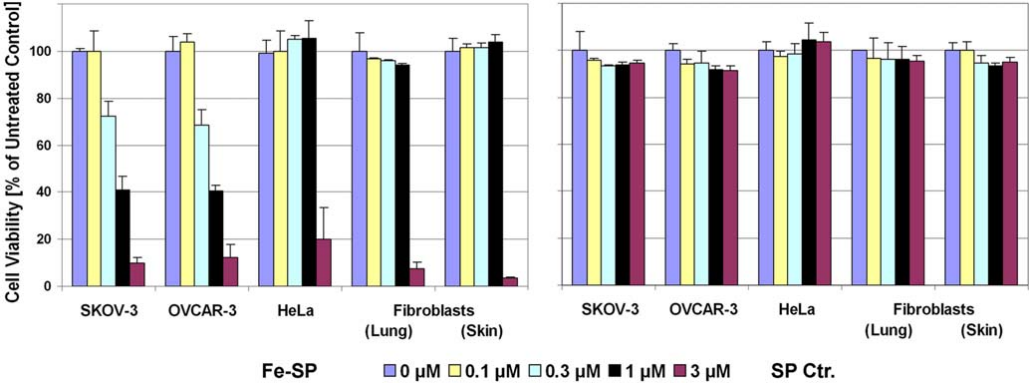
Comparison of the cytotoxic effect of Fe-SP on ovarian cancer and control cell lines. The cytotoxicity of Fe-SP on human ovarian cancer cells (SKOV-3, OVCAR-3) was compared to the effect on HeLa cells or primary fibroblasts at passages 11 to 13 (LF1, human lung; BJ, human foreskin). The cells were treated for a total of 24 h with various concentrations (0.1–3 µM) of Fe-SP or non-complexed SP (Ctr). The MTS viability assay was carried out as described (Materials and Methods). Experiments were performed in triplicates; data are expressed as the mean of the triplicate determinations (X±SD) of a representative experiment in % cell viability of untreated cells [ = 100%].

In an initial approach to analyze the effects of Iron(III)-salophene (Fe-SP) on ovarian cancer cells we performed a viability assay employing SKOV-3 and OVCAR-3 (human platinum-resistant ovarian epithelial adenocarcinoma) cell lines in comparison to HeLa (epithelial cervix adenocarcinoma) or primary lung (LF) or skin fibroblasts (BJ) (passages 10–13). The cells were treated for 24 h with various concentrations (0.1–3 µM) of either Fe-SP or non-complexed salophene (SP) as an additional control to untreated cells. Treatment with SP did not affect the viability of any of these cell lines in the range of 100 nM to 3 µM (Fig 2) nor at concentrations as high as 60 µM (unpublished data). Fe-SP, at concentrations ≥3 µM, exerted highly cytotoxic effects on all cell lines. Remarkably, the response of these cells to Fe-SP was not only dose-dependent but at concentrations below 3 µM cell type specific (Fig. 2). While Fe-SP at concentrations between 300 nM and 1 µM proved to be highly cytotoxic to SKOV-3 and OVCAR-3 cells, treatment of HeLa cells or primary fibroblasts with 1 µM Fe-SP did not result in any change of viability. Thus, Fe-SP depending on the cell line treated, displays selective cytotoxicity, with a dose-dependent response shown here for two platinum-resistant ovarian cancer cell lines.

### Selective morphological changes and induction of apoptosis in SKOV-3 ovarian cancer cells after Fe-SP treatment

**Figure 3:**
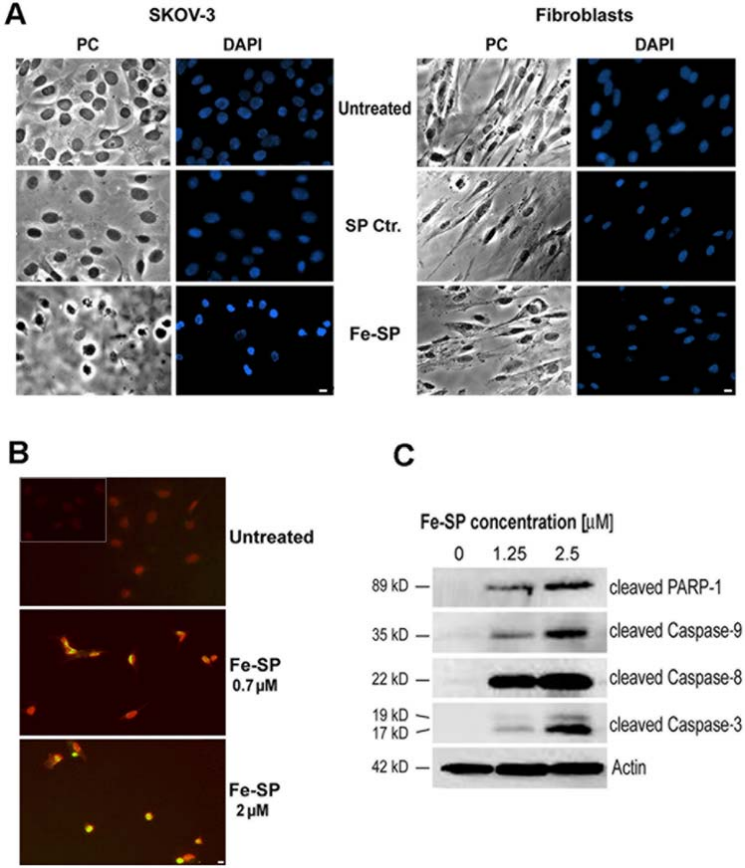
Morphology changes, DNA fragmentation and Caspase activation of ovarian cancer cells and after Fe-SP treatment. (A) Morphological appearance/DAPI staining. Ovarian cancer cells (SKOV-3) or primary fibroblasts (BJ) were treated for 24 h with Fe-SP or non-complexed SP (Ctr.) at a concentration of 2 µM before microscopic analysis by phase contrast (PC) or fluorescence analysis after chromatin staining (DAPI) as described (Materials and Methods). Images obtained from a representative experiment are shown. Bar = 10 µm. (B) Analysis of DNA Fragmentation in a TUNEL Assay. SKOV-3 cells were treated with Fe-SP (0, 0.7, 2 µM) for 24 h. A TUNEL assay was carried out by co-staining with fluorescein-12-dUTP (labeling of DNA nicks in apoptotic cells) and of chromatin with propidium iodide (Materials and Methods). Co-staining of untreated SKOV-3 cells before fixation and permeabilization served as negative control (insert top panel). During fluorescent microscopy, representative images were taken, apoptotic stain (green) and nuclear stain (red) overlaid. TUNEL positive nuclei due to DNA fragmentation appear as yellow areas. Bar = 10 µM. (C) Western Blot Analysis of Caspase activation. SKOV-3 cells were treated with Fe-SP (0, 1.25 µM, 2.5 µM) for 24 h. PAGE and Western blot analysis of cell lysates was carried out. Activated Caspase-3, -8, -9, and inactivated PARP-1 was visualized by immunoblotting using primary antibodies solely recognizing cleaved fragments, not full length pro-forms, of these proteins in combination with a chemiluminescence detection system as described in (Materials and Methods). A representative experiment is shown. As an internal standard for equal loading (50 µg total cell protein/lane) blots were probed with an anti-β-Actin antibody.

To analyze morphological changes of SKOV-3 ovarian cancer cells and primary fibroblasts (BJ) upon Fe-SP treatment we carried out light- and fluorescence microscopy after fixation of the cells and staining of the nuclear chromatin with DAPI. The population of untreated SKOV-3 or cells treated with 2 µM of non-complexed SP for 24 h displayed a homogenous morphology with nuclei lightly and evenly stained by DAPI (Fig. 3A) and occasional appearances of dividing cells in mitosis (bright blue staining of chromosomes lined up along the metaphase plate; not shown here). In contrast, after treatment with 2 µM Fe-SP the majority of SKOV-3 cells displayed shrinkage, highly condensed chromatin, and often densely stained nuclear granular bodies (“apoptotic bodies”) within fragmented nuclei, (Fig. 3A). Primary fibroblasts treated with Fe-SP or SP at the same concentration did not reveal major changes in morphology nor apparent hallmarks of apoptosis showing the selective response of the ovarian cancer cell line studied to Fe-SP.

A common method for detecting DNA fragmentation in the light of cellular apoptotic events is a TUNEL assay, which we employed after treatment of SKOV-3 with Fe-SP. The assay relies on the presence of nicks in the DNA of apoptotic and some necrotic cells, which can be identified by terminal transferase that will catalyze the addition of labeled dUTP (here: fluorescein). SKOV-3 cells were treated with either 0.7 or 2 µM Fe-SP for 24 h. To identify cell nuclei, counterstaining with propidium iodide (Pi), which intercalates in the DNA, was carried out. TUNEL-positive nuclei were identified by yellow spots resulting from an overlay of the image with apoptotic stain (FL-dUTP) and nuclear stain (Pi). As shown (Fig. 3B) no cells before treatment (top panel), 60% of the population of cells treated with 0.7 µM (middle panel) and all cells at 2 µM Fe-SP (bottom panel) were TUNEL-positive cells indicating fragmented DNA.

To define the cellular response of SKOV-3 cells upon Fe-SP treatment we analyzed the activation of caspases characteristic for induction of apoptosis as well as the inactivation of PARP-1 by immunoblotting. Treatment of SKOV-3 cells with 2.5 µM Fe-SP for 24 h resulted in strong activation/cleavage of caspase-9, -8, and -3, while PARP-1 was inactivated/cleaved after drug treatment (Fig. 3C). Treatment with 1.25 µM Fe-SP revealed a similar result, with some activation of caspase-9, and -3, and inactivation of PARP-1 while the activation of caspase-8 was as strong as observed for 2.5 µM Fe-SP. Apparently, the onset of caspase activation and PARP-1 inactivation in SKOV-3 ovarian cancer cells by Fe-SP resulted in the morphological hallmarks of apoptosis observed (Fig. 3A).

### Anti-proliferative effect and cell cycle arrest after treatment of SKOV-3 ovarian cancer cells with Fe-SP

**Figure 4:**
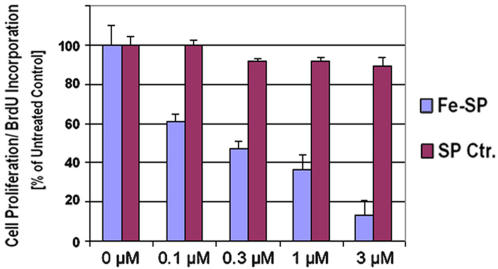
Fe-SP inhibits proliferation of ovarian cancer cells. Ovarian cancer cells (SKOV-3) were treated with various concentrations (0.1–10 µM) of Fe-SP or non-complexed SP (Ctr.) for 24 h. A colorimetric assay (based on BrdU incorporation detected by a BrdU-antibody peroxidase conjugate) was carried out as described (Materials and Methods). The color intensity at 450 nM correlates directly to the amount of BrdU incorporated into the DNA, which in turn represents proliferation. Experiments were performed in triplicates; data are expressed as the mean of the triplicate determinations (X±SD) in % of absorbance by triplicate samples of untreated cells [ = 100%].

As described in the previous sections Fe-SP is a selective cytotoxic drug (Fig. 2) which activates apoptotic processes (Fig. 3) in SKOV-3 ovarian cancer cells. To investigate if Fe-SP exerts anti-proliferative effects and disturbances of cell cycle progression we performed a BrdU incorporation assay. Fe-SP treatment for 24 h dose-dependently reduced cell proliferation (Fig. 4) with an IC_50_ value of 300 nM. In contrast, non-complexed SP did not reduce SKOV-3 proliferation significantly even at concentrations of 3 µM. At the sub-cytotoxic drug concentration of 100 nM Fe-SP (see viability, Fig 2) BrdU incorporation into the DNA was reduced by 40% (Fig. 4).

**Figure 5:**
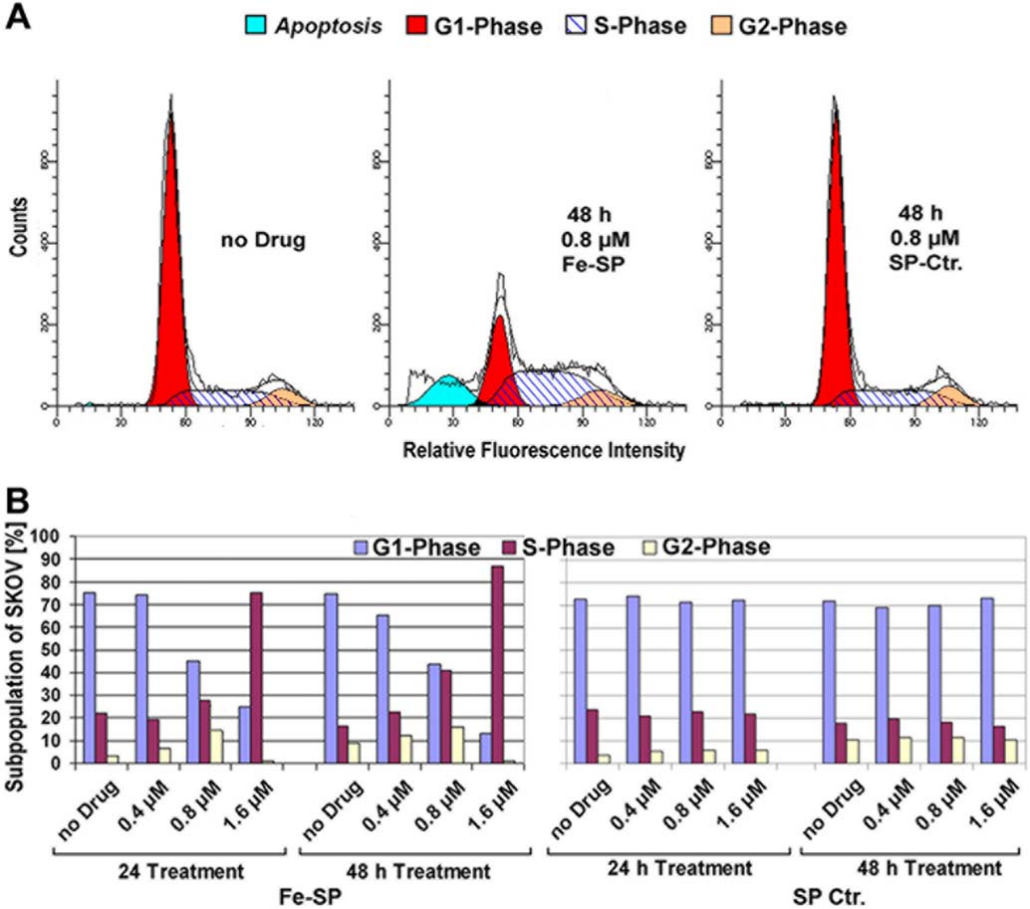
Fe-SP causes S-Phase cell cycle arrest in ovarian cancer cells. Ovarian cancer cells (SKOV-3) were treated with 0, 0.4, 0.8 and 1.6 µM Fe-SP or SP (Ctr.) for 24 or 48 h. Cell cycle analysis by FACS based on propidium-iodide intercalation into the cellular chromatin was carried out as described (Materials and Methods). Data are presented as (A) relative fluorescence intensity in a 2-dimensional FACS profile (ModFit LT software; black lines = data line and model fit line of entire population; shaded areas = model components/subpopulations of G0/G1, S, G2/M, apoptotic cells), example shown for 48 h treatment with 0.8 µM compound, or as (B) comprehensive bar diagram of all data. Standardized gating was used for all samples. Ten thousand events were analyzed for each sample.

In addition to the cell proliferation assay, cell-cycle analysis of propidium iodide stained SKOV-3 cells by flow cytometry was carried out. Fe-SP, in contrast to the non-complexed compound, revealed an increase in the count of sub-diploidal/2n cells (sub-G0/G1, Fig. 5A) in a time- and dose-dependent manner (Table 1). With respect to the cycling cells, Fe-SP causes a time- and dose-dependent arrest of SKOV-3 in S-phase and, thus, a decrease of cells in G0/G1 phase: Untreated cells or SKOV-3 treated with non-complexed SP in this non-synchronous population while cultured *in vitro* are ∼71–75% in G0/G1 and ∼21–24% in S phase (Fig. 5B, Table 1). Treatment with Fe-SP at increasing concentrations (0.4, 0.8, 1.6 µM) for 24 or 48 h caused an increase of cells in S-phase peaking at 75% (1.6 µM/24 h) and 87% (1.6 µM/48 h) of the total population (Fig. 5B, Table 1). Accordingly, the number of cells in G0/G1 decreases to 25% (1.6 µM/24 h) and 13% (1.6 µM/48 h) of the total population, while at this Fe-SP concentration no more cells in G2/M can be detected. At a concentration of 0.8 µM Fe-SP for 24 h and 48 h an increase of cells in G2/M (23%/24 h; 16%/48 h) over the base level (3%/24 h, 9%/ 48 h) can be observed which indicates a delay of this phase along with a developing arrest of cells in S-phase.

**Table 1:** Cell Cycle analysis of SKOV-3 after Fe-SP treatment.

			Fe-SP				SP	Ctr.	
					*Sub-*				*Sub-*
		G0/G1	S	G2/M	*G0/G1*	G0/G1	S	G2/M	*G0/G1*
		[%]	[%]	[%]	[%]	[%]	[%]	[%]	[%]
	***No Drug***	75.0	22.1	2.9	1.2	72.5	24.0	3.5	1.2
**24 h**	***0.4*** **** ***µM***	73.8	19.4	6.9	7.2	73.8	20.9	5.3	0.8
	***0.8*** **** ***µM***	44.7	28.0	13.9	5.8	71.2	22.9	5.9	3.9
	***1.6*** **** ***µM***	24.6	75.4	0	16.7	72.2	21.7	6.1	0.5
	***No Drug***	74.4	16.5	9.0	0.3	71.6	17.8	10.7	0.3
**48 h**	***0.4*** **** ***µM***	65.5	22.3	12.3	9.8	68.9	19.8	11.3	0.7
	***0.8*** **** ***µM***	43.3	40.9	16.3	17.3	70.0	18.4	11.6	1.5
	***1.6*** **** ***µM***	12.9	87.1	0	15.8	73.0	16.4	10.6	1.0

Cell cycle analysis was carried out as described (Materials and Methods). SKOV-3 cells were treated with 0, 0.4, 0.8 and 1.6 µM Fe-SP or SP (Ctr.) for 24 or 48 h. Data were obtained by FACS analysis based on propidium-iodide intercalation into cellular chromatin.

### Cytotoxicity of Fe-SP in rats as a model system

To investigate if Fe-SP causes systemic toxicity *in vivo* we chose rats as a model system. In preliminary the chemotherapeutic relevant doses of Fe-SP in an animal ovarian cancer cell model were determined to be ≤1 mg/kg body weight (Lange et al. in preparation). In these trial studies rat ovarian cancer cells (NUTU-19) were intra-peritoneally (IP) injected into rats and 3 weeks after development of tumors (to mimic the situation following cytoreductive surgery) animals were treated daily with Fe-SP via IP injections. Duration of treatment lasted 12 days and was based on tumor burden in the control animals. Animals were monitored for any discomfort and pain per IACUC protocols. While control animals showed a consistently elevated amount of hemorrhagic ascites, the Fe-SP (1 mg/mL) treated animals displayed significantly less hemorrhagic ascites volume, and less omental tumor weight. Moreover, in this preliminary treatment trial, we observed a complete response in 40% of treated animals (10 rats treated).

Consequently, we carried out 28-day chronic toxicity studies (OECD guidelines for the testing of chemicals (Section-4, No.-407)) in albino rats with Fe-SP concentrations ranging from 0.25 mg/Kg to 4 mg/Kg body weight. This study was designed to investigate the toxicological effects of repeated IP administration of Fe-SP (in DMSO:distilled water at the ratio of 1∶4 on 5 days/week for a period of 28 days). Animals in a low dose group (0.25 mg/Kg body weight), intermediate dose group (1.0 mg/Kg body weight), and high dose group (4.0 mg/Kg body weight) did not reveal any pathological changes as compared to the control group of animals. No mortality or toxic symptoms were observed in test and control groups of animals except ruffled fur in the high dose group. No significant differences were observed in the body weight gain/loss pattern, organ weight, hematological or biochemical parameters (see Material and Methods) of all the test groups when compared to the control group. All parameters fell within the accepted limits of normal variations for albino rats. Microscopic examination of histopathological slides from the low, intermediate, and high dose groups did not show any significant changes except mild degeneration of hepatocytes in three animals (at the high dose) and pulmonary mononuclear cell infiltrates in two animals (high dose).

## Discussion

A low 5-year overall survival rate of only 53% for woman suffering from ovarian cancer is related to the development of resistance of tumor cells to standard chemotherapeutic agents, most notably platinum analogs and therefore new anti-cancer drugs need to be developed [6], [7], [8]. For the present report we chose to study the potential of a novel organometallic complex, Iron(III)-salophene (Fe-SP) as a candidate agent for treatment of ovarian cancer. For purposes of clarification, it is mentioned that the term salophene, more than a century ago, has been applied to 4-acetamidophenyl salicylate, an analgesic, and antipyretic drug manufactured since the early 1900's by Bayer Pharmaceutical Products (Leverkusen, Germany) [14] and is not related to “Salophen” discovered as a superoxide scavenger in E. coli when complexed with manganese(III) [15] that resembles the organometallic compound studied in the present report.

To date no research on the specific effects of salophenes on the viability of cancer cells has been published. In an earlier study on the cytotoxic effects of bicyclic aryl thiazolines on various human cancer cell lines, we determined the range of the colorimetric cell-viability assay used by employing Iron(III)-salophene (not related to thiazolines) at 60 µM as a negative control [16]. Subsequent comparative studies on the effect of transition-metal salophenes (Zn(II)-, Mn(II)-, Cu(II)-, Co(III), Fe(III)-SP) and non-complexed salophene (SP) at lower concentrations revealed that below a concentration of 10 µM only Fe(III)-SP displayed significant cytotoxic effects in various cancer cell lines (Lange et al., in preparation). To test the selective cytotoxic potential of Fe-SP on ovarian cancer cells, we chose two cell lines that are multi-drug resistant alongside with primary lung or skin fibroblasts cell lines. OVCAR-3 cells (ovarian epithelial adenocarcinoma) are resistant to clinically relevant concentrations of adriamycin, melphalan and cisplatin. SKOV-3 cells (ovarian adenocarcinoma) are resistant to several cytotoxic drugs including Cisplatin and Adriamycin (see ATCC, Manassas, VA; www.atcc.org). Similar to the these two ovarian cancer cell lines, the control cells used in the present report, primary fibroblast at early passages and HeLa cells, possess a high metabolism and growth rate. We show here that Fe-SP displayed selective and dose-dependent cytotoxicity against SKOV-3 and OVCAR-3 cells at concentrations between 100 nM and 1 µM, while the viability of HeLa cells (epithelial cervix adenocarcinoma) or primary lung- or skin- fibroblasts (at passages 10–13) at these concentrations was not affected. The relative resistance of the three control cell lines to Fe-SP supported the idea of testing this compound an *in vivo* ovarian animal cancer model. Non-complexed salophene did not affect the viability of either cell line even at concentrations of up to 60 µM nor did 60 µM Fe(III) alone [ferric chloride added] or ferric chloride in combination with non-complexed salophene added to the cell culture media [Lange et al., in preparation] indirectly confirming the stability of the complex and, thus, specific cytotoxic action under cell culture conditions.

Given the present state of research in the field of organometallic compounds such as salophenes or salens (related compound, see introduction), we can only speculate from limited sources about the possible mechanism(s) of cytotoxic action of Fe-SP. A remarkable feature of salens, not yet examined for salophenes, is their affinity to a variety of aromatic neutral molecules such as pyridine, pyridine N-oxide, isoquinoline and benzylamine [17], [18]. Salens when complexed with transition metals act as artificial nucleases. Their reactivity in plasmid DNA cleavage assays can be controlled by conjugation or by the type and charge of the central metal ion core [18], [19], [20]. For example, Ni(II)- or Mn(II)-salens were found to efficiently induce DNA strand scission but not Cr(II)- or Cu(II)- [18], [20] or Fe(II)-salens unless additional hydroxyl groups facilitate their oxidation to Cu(III) or Fe(III) species [21]. It has been postulated that Fe-salen in cooperation with the quinine system facilitates the formation of Iron(III)^.^O_2_^−^ species to produce free hydroxy radicals responsible for DNA cleavage [21]. However, the DNA cleavage activity of Fe(III)-salen is higher than of Fe(II)-salen suggesting that the increased water-solubility and charge of the Fe(III) ion is dominating the cleavage potential of the structure [22] which likely applies to iron(III)-salophene, as well. In addition, the authors linked the DNA cleavage activity by Fe(III)-salen *in vitro* to the observation that this compound caused nuclear fragmentation and induction of apoptosis (via mitochondrial pathway/cytochrome c release) in HEK293 cells at a concentration of 50 µM [22].

While the mechanism(s) of action of salens or Fe-SP on cells in culture or *in vivo* remains to be investigated, selective morphological changes of cancer cell lines and induction of apoptosis after drug treatment *in vitro* are a first indicator for potential selective effects on tumor metastasis and cell physiology *in vivo*. The present study shows that Fe-SP at concentrations of 2 µM in SKOV-3 ovarian cancer cells but not in primary fibroblasts caused nuclear fragmentation, chromatin condensation, DNA fragmentation which are all classic hallmarks of apoptosis [23]. Western blotting confirmed the activation of effector caspase-3 and inactivation of PARP-1 (involved in DNA repair) [24] during the execution of apoptosis in SKOV-3 cells following Fe-SP treatment. Moreover, it was apparent that Fe-SP induced both major signaling pathways (*intrinsic, extrinsic*) described for programmed cell death [25], [26]. We observed strong cleavage/activation of initiator caspase-8, typical for the involvement of the *extrinsic* apoptotic pathway [27], [28], upon Fe-SP treatment of SKOV-3 cells. The *intrinsic* pathway mediates apoptotic responses to stress signals such as drugs, DNA damage or growth factor deprivation and is initiated by mitochondrial damage. It results in the mitochondrial release of cytochrome C which activates initiator caspase-9 [28], [29] and was shown to contribute to cell death of HEK293 (human kidney cell line) upon Fe(III)-salen treatment [22] and also in the present study for SKOV-3 cells upon Fe(III)-salophene treatment. To date, no other studies describing the morphology or apoptotic signaling of any cell lines after treatment with compounds belonging to the class of iron-salens or -salophenes have been published.

Similarly, no publications examining the change in cell cycle progression following treatment with either a salen or salophene metallocomplex exist. While one publication observed a change in viability and morphology of skin fibroblasts in culture after treatment with a Cr(III)-salen complex at high concentrations of 50 µM, the following cell cycle studies were only conducted with a ethylendiamine-Cr(III)-chloride of higher cytotoxicity [30]. We show here that Fe-SP dose-dependently reduced the proliferation of SKOV-3 ovarian cancer cells (IC_50_ at 300 nM). Even at the sub-cytotoxic concentrations (100 nM Fe-SP) BrdU incorporation into the DNA was reduced indicating that Fe-SP exerts effects as an anti-proliferative and, thus, a potential anti-cancer agent. Additional cell-cycle analysis of SKOV-3 after Fe-SP treatment, as already indicated in the TUNEL assay, revealed an increase in the sub-diploidal population which represents cells with significant DNA damage, indicating a late apoptotic stage. With respect to the cycling cells, Fe-SP at 1.6 µM caused a full arrest of SKOV-3 in S-phase along with decrease of cells in G0/G1 and a total loss of cells in G2/M. Apparently, Fe-SP treatment affected cell-cycle checkpoints in S-phase causing a dose and time-dependent reduction of cell-cycle progression. To date, this is the first report on the regulation of the cycle of any cell line by treatment with either metallosalen or metallosalophene compounds. Furthermore, only few data on the effect of transition metal complexes in general on the cell cycle exist, such as the arrest of a neuroblastoma cell line in G1-phase when treated with an isatin-schiff base copper(II) complex [31].

Even though not the objective of this report, further studies could analyze the effects of Fe-SP on cell-cycle checkpoints in synchronized SKOV-3 cultures and other cancer and non-transformed cell lines. Targeting such checkpoints has been suggested as an alternative approach to anti-cancer therapies [32], [33]. Regulators of the cell-cycle machinery are frequently altered in human cancer and apparently transformed cells can be more sensitive to the cyclin-dependent kinases (CDK) inhibition [34], [35]. With respect to S-phase events both mouse models and studies on cancer-derived cells revealed that accumulation of cyclin D1/CDK4 complexes triggers DNA re-replication [36] and, thus, could be specifically targeted in cancer cells. As one example, Guggulsterone, a plant-derived drug, caused cell-cycle arrest in S-phase by the suppression of cyclin D1 and cdc2 and increased cyclin-dependent kinase inhibitor p21 and p27 expression in a wide variety of human tumor cell types [37]. Based on the specific arrest of SKOV-3 cells by Fe-SP in S-phase observed in the present study, in future studies we will analyze the effects of this novel organometallic compound and potential cancer therapeutic on specific cell-cycle regulators (CDK's, cyclins) and replication-start and -progression signals of the S-phase [38], [39] in platinum-resistant ovarian cancer cells.

To set the stage for further investigation of Fe-SP as a potential therapeutic drug in addition to these *in vitro* studies we conducted a standardized study (28-day chronic toxicity) on the systemic toxicity of Fe-SP when applied in rats as a model system. When intra-peritoneally administered, Fe-SP did not show any systemic toxicity at even four times the concentration (4 mg/Kg body weight) that in our preliminary treatment trials were determined to be chemotherapeutic relevant doses (≤1 mg/Kg body weight) of Fe-SP in a rat ovarian cancer cell model. The present report suggests that Fe-SP is a potent growth-suppressing agent *in vitro* for cell lines derived from ovarian cancer and a potential therapeutic drug to treat such tumors *in vivo*.

## Materials and Methods

Animal experiments were carried out in the animal facilities of Rhode Island Hospital (RIH), RI USA with strict adherence to the guidelines of the Animal Welfare Committee of RIH and Women & Infants Hospital and at the Shriram Institute for Industrial Research, Delhi, India in accordance with the guidelines set by the Laboratory Animal Safety Committee of the Shriram Institute.

### Synthesis of Iron(III)-salophene

The synthesis of the salophene ligand (SP, Fig. 1) followed a previously reported procedure [40]. 1,2-phenylenediamine (1.24 g, 11.5 mmol) was added to a solution of *o*-vanillin (3.5 g, 23 mmol) in anhydrous ethanol (20 mL) and the yellowish solution stirred and refluxed for 2 h. The reddish suspension was filtered and re-crystallized with boiling ethanol and the resultant reddish needles collected and dried (2.8 g SP, 65% yield). The Iron(III)-salophene complex (Fe-SP, Fig. 1) was prepared as follows: SP (550 mg, 1.46 mmol) dissolved in acetone (44 mL) was carefully combined with ferric chloride hexahydrate (394.9 mg, 1.46 mmol) dissolved in acetone (5.5mL). The resultant microcrystalline solid that grew in solution was filtered, washed thoroughly with acetone, and dried to obtain the Fe-SP complex (244.6 mg, 35% yield). Fe-SP was dissolved in DMSO (dimethyl sulfoxide) for experiments in tissue culture and characterized by HPLC and X-ray crystallography (Table 2) with a high resolution x-ray diffraction system (Bruker SMART diffractometer using SHELXTL-97 software; Bruker AXS Inc., Madison, WI).

**Table 2:** Crystal data and structure refinement of Fe-SP (crystallized in acetone)

**Identification code**	kko133cmsad	
**Empirical formula**	C_25_H_26_ClFeN_2_O_6_	
**Formula weight**	541.78	
**Temperature**	90(2) K	
**Wavelength**	0.71073 Δ	
**Crystal system**	Monoclinic	
**Space group**	P2(1)/n	
**Unit cell dimensions**	a = 10.2706(12) Δ	α = 90°
	b = 13.9248(17) Δ	α = 101.104(2)°
	c = 16.985(2) Δ	α = 90°
**Volume**	2383.7(5) Δ^3^	
**Z**	4	
**Density (calculated)**	1.510 Mg/m^3^	
**Absorption coefficient**	0.790 mm^−1^	
**F(000)**	1124	
**Crystal size**	0.30×0.30×0.08 mm^3^	
**Theta range for data collection**	1.91 to 28.31°.	
**Index ranges**	−13< = h< = 13, −18< = k< = 18, −22< = l< = 22	
**Reflections collected**	20658	
**Independent reflections**	5769 [R(int) = 0.0389]	
**Completeness to theta = 28.31°**	97.4%	
**Absorption correction**	Empirical	
**Max. and min. transmission**	0.9 and 0.8	
**Refinement method**	Full-matrix least-squares on F^2^	
**Data/restraints/parameters**	5769/0/322	
**Goodness-of-fit on F^2^**	1.046	
**Final R indices [I>2sigma(I)]**	R1 = 0.0420, wR2 = 0.0997	
**R indices (all data)**	R1 = 0.0536, wR2 = 0.1065	
**Largest diff. peak and hole**	0.710 and −0.311 eΔ^−3^	

### Cell culture

SKOV-3 (human ovarian adenocarcinoma), OVCAR-3 (human ovarian epithelial adenocarcinoma), HeLa (human epithelial cervix adenocarcinoma), LF1 (primary human lung fibroblasts), and BJ (primary human fore skin fibroblasts) cell lines were obtained from American Type Culture Collection (Manassas, VA). Cells were grown in T75 cell culture flasks (Corning, New York, NY) to a confluency of ∼80% in complete Dulbecco's Modified Eagle's Medium (DMEM) supplemented with sodium ascorbate (50 µg/ml), glutamine (300 µg/mL), penicillin (400 µg/mL), streptomycin (50 µg/mL) and 10% fetal calf serum (FCS) on in a humidified atmosphere of 5% CO_2_ and 95% air at 37°C. For all assays cells, after seeding, were allowed to attach overnight in complete medium before treatment as indicated in complete medium.

### Cell viability assay

Viability of cell lines before and after drug treatment was determined by the CellTiter 96® AQueous One Solution Assay (Promega Corp, Madison, WI) following the manufacturer's recommendations. This colorimetric assay is based on the ability of mitochondria to reduce a substrate [MTS, 3-(4,5-dimethylthiazol-2-yl)-5-(3-carboxymethoxyphenyl)-2-(4-sulfophenyl)-2H-tetrazolium] into a soluble formazan product quantified by measuring the absorbance at 490 nm. The resulting OD is directly proportional to the number of living cells [41]. Briefly, cells (5×10^3^/well) were plated into 96 well flat bottom plates (Corning, Inc., Corning, NY) before treatment with various drugs or vehicle (DMSO) as indicated. Following incubation at 37°C in a cell culture incubator for 20h MTS reagent was added at a 1∶10 dilution to the medium. The samples were incubated for an additional 4 h before absorbance was measured at 490 nm in an ELISA plate reader (Thermo Labsystems, Waltham, MA). Experiments were performed in triplicates; data are expressed as the mean of the triplicate determinations (X±SD) of a representative experiment in % of absorbance by samples with untreated cells [ = 100%].

### Morphological Studies

Cells were seeded (1×10^4^/chamber) into a Lab-Tek Chamber-Slide System (Nalge Nunc., Naperville, IL) and treated for 24 h with 2 µM Fe-SP or SP as a negative control alongside with non-treated cells. Following two wash-steps in PBS the cells were fixed in PBS, 2% PFA, 0.2% Triton X for 20 min at RT and stained for 10 min with 200 ng/mL 4′-6-Diamidino-2-Phenylindole (DAPI) in PBS before mounting. Representative images were taken with an inverted microscope (Nikon Eclipse TE2000-E, CCD camera) and 20x objective.

### TUNEL Assay

DNA fragmentation was detected using the DeadEndTM Fluorometric TUNEL System assay (Promega, Madison, WI) according to the manufacturer's recommendations. Cells (5×10^3^/well) were plated into 96 well flat bottom plates (Corning, Inc., Corning, NY), treated with 0.7 or 2 µM Fe-SP and the assay carried out as described previously [42]. Fluorescence of apoptotic cells (green; labeling of DNA nicks by fluorescein-12-dUTP) and of chromatin (red; staining of chromatin with propidium iodide) was detected by fluorescence microscopy with an inverted microscope (Nikon Eclipse TE2000-E) and a 10x objective. Four randomly chosen microscopic fields were captured.

### Western blot analysis

Cells were seeded into 100 mm^2^ tissue culture dishes (5×10^5^ cells/dish), and treated with 1.25 or 2.5 µM Fe-SP for 24 h. Preparation of cell lysates, PAGE and immunoblotting was carried out as described previously [43]. Primary antibodies purchased from Cell Signaling Technology (Beverly, MA) against cleaved Caspase-3 (#9661), cleaved caspase-8 (#9748), cleaved caspase-9 (#9501), cleaved PARP-1 (#9541), β-actin (#1501) were used. Antibodies were diluted 1∶1000 in 5% BSA/PBST; incubation and wash steps carried out as in [43]. The antibody specific for cleaved caspase-3 detects the large fragment (17/19 kD) of activated caspase-3 (cleavage adjacent to Asp175) and does not recognize the full length protein. The antibody specific for cleaved caspase-8 detects the small fragment of caspase-8 (cleavage at aspartic acid384) and does not cross-react with the full length protein. The antibody specific for cleaved caspase-9 detects the large fragment of caspase-9 (cleaved at Asp330) and does not recognize full length protein. The antibody specific for cleaved PARP detects the large fragment of PARP-1(produced by caspase cleavage at Asp214) and does not recognize full length PARP-1 or other PARP isoforms. The bands were visualized using horseradish peroxidase-conjugated secondary antibodies (Amersham-Pharmacia Biotech, Piscataway, NJ), followed by enhanced chemiluminescence (Upstate, Waltham, MA) and documented by autoradiography (F-Bx810 Film, Phenix, Hayward, CA).

### Cell Proliferation Assay

Proliferation of various cell lines was determined by a BrdU (5-bromo-2′-deoxyuridine) incorporation assay (Roche Applied Science, Indianapolis, IN) according to the manufacturer's recommendations. Briefly, cells (5×10^3^/well) were plated into 96 well flat bottom plates (Corning, Inc., Corning, NY) before treatment as indicated (result section) for 18 h in FCS free media. BrdU (10 µM final concentration) was added to the cells grown for further 6 h. After washing cells were fixed and incubated for 2 h at 37°C with an anti-BrdU antibody-peroxidase conjugate. Immunocomplexes were detected by addition of a tetramethyl-benzidine (TMB) substrate solution according to the manufacturer's recommendations. The reaction was stopped by adding 50 µL of 1M sulfuric acid, and the absorbance was measured with an ELISA plate reader (Thermo Labsystems, Waltham, MA) at 450nm. In this assay, the color intensity correlates directly to the amount of BrdU incorporated into the DNA, which in turn represents proliferation. Experiments were performed in triplicates; data are expressed as the mean of the triplicate determinations (X±SD) of a representative experiment in % of absorbance by samples with untreated cells [ = 100%].

### Cell Cycle Analysis

Cell cycle analysis and quantification of apoptosis was carried out by flow cytometry. Cells were seeded into 100 mm^2^ tissue culture dishes (7.5×10^5^ cells/dish) and treated with Fe-SP as indicated (result section) At the end of the incubation period detached cells were collected in 15 mL polypropylene centrifuge tubes along with the medium; culture dishes were washed once with PBS. Adherent cells were scraped off and combined in the same tube. After centrifugation (250 g, 5 min), cells were fixed and permeabilized with ice-cold 70% ethanol for 30 minutes, followed by incubation with 50 µg/mL of propidium iodide and 100 µg/mL of RNase for 30 min at 37°C in the dark. Data was acquired on a BD FACSort flow cytometer using CellQuest software (BD Immunocytometry Systems, San Jose, CA) and analyzed using ModFit LT software (Verity Software House, Inc., Topsham, ME). Ten thousand events were analyzed for each sample. Appropriate gating was used to select the single cell population and used on all samples, ensuring that the measurements were made on a standardized cell population.

### *In vivo* toxicity study

#### Sample preparation

Working solutions of Fe-SP from a stock solution of 10 mM in 100% DMSO were prepared in vehicle (DMSO:distilled water at a ratio of 1∶4) for repeated intraperitoneal administration of rats (0.25 mg/Kg, 1 mg/Kg, and 4 mg/Kg body weight).

#### Animals

Thirty healthy adult male and thirty healthy adult female rats (Wistar-albino rats, 6–8 weeks of age and weighing 160–180 g, purchased from the laboratory animal facility of the Shriram Institute, Delhi, India) were acclimatized for 7 days and animals were caged in a group of 5 according to sex in polypropylene cages fitted with wire mesh tops and having sterilized paddy husk bedding. The rats were then randomized and assigned to 6 groups of 5 male and 5 female rats each and identified by cage tag having name of test substance and details of group, dose level and sex.

#### Administration

The first group, i.e. the control group, was given vehicle only. The second, third, and fourth groups of animals were administered 0.25, 1.0, or 4.0 mg/Kg body weight of Fe-SP in vehicle. The fifth and sixth groups were assigned as the satellite control and satellite high dose group (4.0 mg/Kg body weight) and treated with vehicle only (control) or Fe-SP in vehicle. The rats were observed daily for behavior, appearance, and toxicological sign and symptoms.

#### Body weight

Recorded individually before treatment and at weekly intervals, thereafter group mean body weights were calculated.

#### Signs/symptoms

Macroscopic evaluation, recorded daily in terms of clinical manifestation, if any.

#### Termination of the study

The following clinical laboratory determinations were made in all the animals of each group after termination of the experiment. Feed was withdrawn overnight prior to the collection of samples. Four to 6 ml of blood was withdrawn by cardiac puncture under light nembutol anesthesia prior to sacrifice. The following hematological measurements were performed on control and treated groups of animals (using a Baker Hematology system 9120+): WBC, RBC, Hemoglobin (Hb), Hematocrit (Hct), Platelets, Neutrophils (N), Lymphocytes (L), Basophils (B), Monocyte (M), Eosinophils (E), and Prothrombin time. The following serum chemistries were also performed on control and treated rats using Boehringer Mannheim diagnostic kits: Blood Glucose, Blood urea nitrogen (BUN), Total protein (TP), Albumin, Serum glutamic oxalo acetate transaminase (SGOT), Serum glutamic pyruvic transaminase (SGPT), Serum alkaline phosphatase (SAP), and Cholesterol.

#### Post mortem evaluation

No mortality during the study in treatment or control groups was observed. After 28 days the following organs from all animals were weighed: brain, heart, kidneys, liver, lungs, spleen, adrenals, testis, ovaries, uterus. Microscopic histopathological examination of the tissues listed above as well as of stomach and intestine were carried out. The student's t-test was used for the biostatistical interpretation of the animal data.
