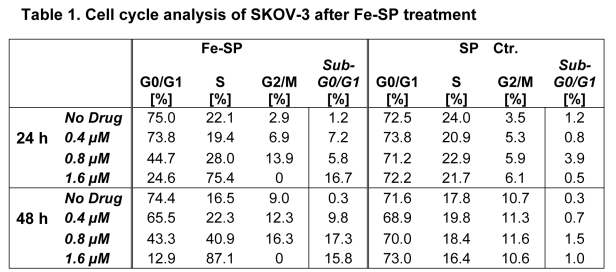# Correction: Iron(III)-Salophene: An Organometallic Compound with Selective Cytotoxic and Anti-Proliferative Properties in Platinum-Resistant Ovarian Cancer Cells

**DOI:** 10.1371/annotation/d97d24fc-aa07-40fd-88b2-6b2e050ddb31

**Published:** 2008-07-01

**Authors:** Thilo S. Lange, Kyu Kwang Kim, Rakesh K. Singh, Robert M. Strongin, Carolyn K. McCourt, Laurent Brard

Table 1 appears incorrectly. Please view the corrected table here:

**Figure:**